# Insomnia among Town Residents in Ethiopia: A Community-Based Cross-Sectional Survey

**DOI:** 10.1155/2019/6306942

**Published:** 2019-05-02

**Authors:** Tilahun Ali, Habte Belete, Tadesse Awoke, Fisseha Zewde, Habtamu Derajew, Solomon Yimer, Melak Menberu

**Affiliations:** ^1^College of Health and Medical Sciences, School of Nursing and Midwifery, Department of Psychiatry, Haramaya University, Harar, Ethiopia; ^2^Psychiatry Department, College of Medicine and Health Science, Bahir Dar University, Bahir Dar, Ethiopia; ^3^Biostatistics Department, College of Medicine and Health Science, University of Gondar, Gondar, Ethiopia; ^4^Amanual Mental Specialized Hospital, Addis Ababa, Ethiopia; ^5^Psychiatry Department, College of Medicine and Health Science, Dilla University, Dilla, Ethiopia

## Abstract

**Background:**

Insomnia is one of the most common sleep problems throughout the world and a major public health concern among adults in the general population. The aim of this study was to assess the prevalence of insomnia and its associated factors among town adult residents in Ethiopia.

**Methods:**

Community-based cross-sectional study was done among 840 randomly selected adult participants by using standardized and pretested Athens insomnia scale (AIS) to assess insomnia. Systematic random sampling technique was used to get samples of the study participants. Data were entered into Epi-Info and analyzed using SPSS version 20. Descriptive, bivariate, and multivariate logistic regression models were used for analysis. Adjusted odds ratio (AOR) with 95% Confidence Interval (CI) was used to show the odds, and P value < 0.05 was considered as statistically significant.

**Results:**

The prevalence of insomnia was found to be 42.9%. Sleep problems were associated with female sex [AOR =2.74, 95% CI; (1.77, 4.24)], age above 48 years [AOR=4.67, 95% CI: (2.32, 9.40)], being single [AOR=2.81, 95% CI (1.59, 4.95)] and widowed [AOR=4.20, 95% CI; (1.60, 11.01)], khat chewing [AOR=1.76,95% CI; (1.19, 2.60)], current tobacco smoking [AOR=3.13, 95% CI; (1.64, 5.95)], caffeinated beverage use [AOR=1.67, 95% CI; (1.12, 2.49)], comorbid medical-surgical disorders [AOR=2.03, 95% CI; (1.18, 3.48)], common mental disorders [AOR=8.92, 95% CI; (5.93,13.44)], and noise at bed time [AOR=2.13 95% CI; (1.20, 3.78)].

**Conclusion:**

The prevalence of insomnia has to be found high and associated with many area related factors. It is important to pay attention in urban settings and large scale studies recommended.

## 1. Introduction

Sleep is a basic and necessary biological process in which crucial for physical and mental functioning. In our modern society, people have face difficult of sleep and insomnia is one major complain in the individual life. Insomnia is characterized by difficulty falling asleep or staying asleep in adults. According to the Diagnosis and Statistical Manual of Mental Disorders fifth edition (DSM-5), sleep-wake disorders are broadly classified into eleven. Insomnia disorder is the one and the first concern; other sleep-wake disorders included in the topic are parasomnias, narcolepsy, and hypersomnolence disorder [1–3]. In our modern society sleep deprivation is the most important public health concern and affects more than 150 million people in the developing world [4, 5]. The negative health outcome of sleep problems is observed in day to day functioning of individuals with insomnia. People with insomnia have disturbances in cognitive and psychomotor function in which negatively influence individual's wellbeing, productivity, and safety. Insomnia has been associated with increased morbidity and mortality that incur financial costs [6, 7].

Even though the etiological aspect of sleep problems is numerous and complex many scholars briefly express that factors like mental illness, medical/neurological conditions, environmental factors, sociodemographic characteristics, stressful life events, and substance use are involved as risk factors of insomnia [1, 8–12]. Insomnia is a widespread public health problem that affects many individuals' wellness and increase the risk to injuries, but most of the time individuals who are suffering from insomnia will not report their problem. This indicates insomnia is a major public health problem which is not appropriately addressed in the community [13].

Many studies around the world reports on insomnia in different populations, for instance, in South Korea; insomnia in the general population was 17% and, in Nigeria, it was 27.5% among geriatric population [9, 14]. From multicenter cross-sectional study among eight low income Africa and Asia countries, sleep difficulties was found 16.6%, with a great variation across countries with range from 3.9% to 40.0% [5].

Insomnia is not exclusively selective and it affects the entire population with a wide range of magnitude throughout the world. The magnitude of insomnia in a community survey in Tennessee, USA, was 19.7% [15], in China 39.4% [16], in United Kingdom 37% [17], in Sweden 10.5% [18], in Portugal 46.8% [19], in Canada 21.4% [20], in west Bengal, India, 15.4% [21], in Korea 22.8% [22], in a longitudinal study in Canada 15.4% [23], in Greece 25.3% [24], in Turkey 15.3% [25], in Karachi, Pakistan, 31.3% [26], in Egypt 33.6% [27], and in Nigeria 27.5% [9].

However, there is no previous study related to sleep problems in Ethiopian and this report will be the first informative study to the country. Therefore, the aim of this community survey was to see the magnitude and associated factors of insomnia in Ethiopian urban residents.

## 2. Methods

Community-based cross-sectional study was conducted in Dessie town from 03 May to 02 June 2016. Dessie is located in South Wollo, Amhara Regional state, Ethiopia, and far from about 401 kilo meters Northern of Addis Ababa. According to the 2007 national population and housing census report, the total population size of Dessie town was estimated to be 120,095 with ten urban subcities. Currently Dessie town has one referral hospital, three private hospitals, and three governmental health centers. Dessie referral hospital has 3 psychiatric outpatient departments. The geographical setting of the town is surrounded by mountains and cross road to the capital city of Ethiopia (Addis Ababa), the Tigray regional city (Mekele), and the Amhara regional city (Bahir dar). There is high consumption of khat in the town. Even though the town is a part of Amhara regional state, the population and cultural asset is mixed by other regional populations like Tigray and Afar regions.

### 2.1. Participants

All permanent adult residents in Dessie town (at least six months of duration) were the source of population whereas those residents who were available during the study period are considered as the study population. Residents who live in Dessie town at least for six month were included in the study and those who cannot give information were excluded.

The number of samples required for the study was estimated by taking 50% of the prevalence of insomnia among adults in a single population proportion for unknown prevalence, with 5% of marginal error, design effect of two, and 10% nonresponse rate. A multistage stage sampling technique was employed for the total of 846. At stage one, from the 10 subcities three subcities were selected randomly using lottery method. Then individual households in the chosen subcities were selected using systematic sampling technique after identifying an initial starting household. The sample sizes were distributed to each subcity proportional to the household size of the subcities and adults in the selected household were further selected and interviewed. In the case of more than one eligible participant in the household, lottery method was used to select only one in the family. The total household in the selected subcity was 3384 and the constant skip interval K-value was 4. Data was collected by degree holder psychiatric nurses by using a semistructured questionnaire.

### 2.2. Data Collection Instruments

Insomnia was detected by using Athens insomnia scale. Those participants who scored 6 and above are considered to have insomnia [28]. The presence of insomnia was assessed using the Athens insomnia scale (AIS). The AIS is 8-item self-reported questionnaire within the past month and scores each ranging from 0 to 3 (0 score indicated better and 3 is worst). AIS has a sensitivity of 93% and specificity of 85% and it is reliable in such study with Cronbach's *α* of (internal consistency) 0.83 [28]. Common mental disorder was considered when adults score 6 or more symptoms of the 20 self-reporting questionnaire (SRQ-20) questions in the last one month [29]. Current substance use was considered when participants use currently substances (khat, caffeine, cigarette and alcohol) in the last three months [30], since those substances are commonly used in the community as licit in the town. The presences of diagnosed mental, medical, or surgical disorder were verified during the interview and other sociocultural variables were included.

Socioeconomic status of the study participants has been assessed using principal component analysis in which eigen values greater than one were used as an extraction and factors to be extracted was fixed to three (from the low, medium, and high). To increase the quality of the data, we use Amharic (local language) version during data collection then translated to English during analysis; training was given for data collectors as well as supervisors and we also use design effect.

### 2.3. Analysis

The data was entered and clean up with Epi-Info version 7 and analyzed by using Statistical Package for Social Science version 20. Descriptive statistic was used to show clear picture of the problem. Multivariate and binary logistic regression analysis was implemented to identify factors associated with insomnia. During binary regression p value at 0.2 was considered for candidate for multivariate. The strength of the association was presented by odds ratio with 95% confidence interval (CI). At multivariate P value less than 0.05 was considered as statistically significant in this study.

### 2.4. Ethical Clearance

Ethical clearance was obtained from Ethical Review Board of University of Gondar and formal permission letters were taken from administrative of the town and written consent taken from the participants. Confidentiality is maintained by omitting personal identification. For those individuals who had insomnia and positive for common mental disorders, we had link them to psychiatric service.

## 3. Results

A total of 846 participants were included in the study and six of them 6 (0.7%) were discontinue their interview by their will during data collection. The remaining 840 participants which make the response rate 99.3% completed the interview. The majority of the respondents were females 503 (59.9%). Of the participants, 469 (55.8%) were orthodox, 828 (98.6%) were Amharic by ethnic group, and 325 (38.7%) were married (Table 1).

### 3.1. Substance Use Related Information

The result of this study revealed the prevalence of current substance use and that 69.3% of them use caffeinated beverage in the last three months from the data collection (Table 2).

### 3.2. Clinical and Medication Use

From all the respondents 169 (20.1%) of them had current medical-surgical disorders including hypertension (77), diabetes mellitus (47), asthma (35), HIV/AIDS (41), and gastritis (49). Among the participants 243 (28.9%) have psychological distress (anxiety, depression, and Somatization) and 232 (27.6%) of the participants use medication for any reason.

### 3.3. Environmental, Leisure Time Physical Activity, and Technological Factors

Among the respondents 127 (15.1%) were disturbed by excessive noise during sleep, 117 (13.9%) suffered from excessive light during sleep, 161 (19.2%) were involved in shift work, 369 (43.9%) were watching television during sleep time, 300 (35.7%) use smart phone close to bed time, and only 89 (10.6%) engaged in leisure time physical activity.

### 3.4. Magnitude of Insomnia

The prevalence of insomnia in this study was 42.9% and factors in the bivariate analysis sociodemographic variables (age, sex, marital status, educational status, and occupation), behavioral factors (current substance use, caffeinated beverage use close to bed time, and leisure time physical activity), clinical factors like current medical-surgical disorders, common mental disorders, and any type of medication use, environmental factors (excessive noise and light), shift work, and using smart phone close to bed time fulfilled the minimum requirement (p<0.2) and were taken for further analysis into multivariate analysis.

After multivariate analysis of insomnia in relation to all independent variables, age, sex, marital status, current medical/ surgical disorders, common mental disorders, current use of substance (khat, tobacco, and caffeinated beverage), and excessive noise at bed time were found to be statistically significant.

The odds of insomnia were nearly threefold among female respondents as compared to males (48.9% versus 33.8%) [AOR =2.74, 95% CI; (1.77, 4.24)]. Prevalence of insomnia was found to be increasing with advancing age. Highest prevalence was observed in the age group of ≥48 years (56.98%) [AOR=4.67, 95% CI: (2.32, 9.40)] followed by 38-47 years [AOR=3.02, 95% CI: (1.47, 6.20)]. Those who are single [AOR=2.81, 95% CI (1.59, 4.95)] and widowed [AOR=4.20, 95% CI; (1.60, 11.01)] by current marital status were more likely to suffer from insomnia more than two and four times, respectively.

Clinical factors like current medical-surgical disorders had more than two times likely to develop insomnia than those who do not have current medical-surgical disorders [AOR=2.03, 95% CI; (1.18, 3.48)]; common mental disorders are strongly associated with insomnia more than eight times [AOR=8.92, 95% CI; (5.93,13.44)].

Concerning the respondents current substance use those who chew khat currently have almost two times more likely to develop insomnia as compared with nonchewers [AOR=1.76,95% CI; (1.19, 2.60)]. Current smokers were more than three times more likely to have insomnia when compared with nonsmokers [AOR=3.13, 95% CI; (1.64, 5.95)]. Caffeine consumption was associated with insomnia with odds of more than one and half [AOR=1.67, 95% CI; (1.12, 2.49)]. Those participants who face excessive noise during the night suffered from insomnia [AOR=2.13 95% CI; (1.20, 3.78)] when compared with those who did not experience (Table 3).

## 4. Discussion

Many scholars agreed that the source of sleep difficulties arises from neurological, behavioral, and medical illnesses in the population. Our findings come with certain behavioral factors like use of coffee and khat chewing (in which common habitual practice in this town) which had significance association to insomnia with other factors. This study was a first attempt to ascertain the prevalence of insomnia and associated factors among Dessie town adults in Ethiopia and it helps the literature by addressing the physical, psychological, and medical aspect of sleep difficulties. Of course, it is pioneer study in this area and perhaps in the country and may help local policy makers and health workers by being the baseline information concerned to insomnia in Dessie town.

The prevalence of this finding was in line with the study done in China which is 39.4% [16], but higher than in UK 37% [17], South Korea 17% [14], Greek 25.3% [24], and Turkey 15.3% [25]. The possible explanation for the difference in prevalence may be due to study population, tools and study setting. However, the finding of this study was slightly lower than that of Portugal's study 46.8% [19] and a study from Poland 50.5% [10].

In this study age was significantly associated with insomnia, in which those age groups from 38-47 and 48 and above years were more than three and four times and more likely to develop insomnia, respectively, as compared with age groups 18-24. This may be due to the fact that insomnia is common in elderly since aging is related highly with comorbidity of medical and surgical illness. This finding was supported by different studies done in Greece [24].

Being female was also associated with insomnia nearly three times more likely to have insomnia as compared to males. The possible reason could be that females may have familial burden (multiple roles and responsibilities), gender discrimination like gender based violence, and comorbid common mental illness such as depression and anxiety which is highly prevalent among females than males. It also explained by hormonal changes during menstruation in women [22]. This finding was supported by studies from China [13], Turkey [25], and Egypt [27].

Marital status was associated with insomnia in which single and widowed participants were more than three times and more than four times more likely to develop insomnia than married ones, respectively. It is in agreement with studies done in China [13]. The possible explanation for this might be feeling of aloof, social stress, and lack of personal relationships with others.

In regard to current substance use, khat use was significantly associated with insomnia with almost twofold more likely to develop insomnia as compared to none khat users. This finding is supported by studies done in Ethiopian college students [8]. Those who smoke cigarette had three times odds of developing insomnia as compared with nonsmokers. Consuming caffeinated beverage close to bed time had one and half times risk of developing insomnia. Possible reasons behind this may be because of the biological effect of the substances and adverse life events due to substance usage are associated with symptoms of insomnia.

Those participants who had current medical disorders like HIV/AIDS, hypertension, diabetes mellitus, and asthma had three times more likely developing insomnia than participants without medical illness. The finding coincides with study from Canada [20], West Bengal India [21], and Egypt [27]. The possible justification for this strong association with insomnia is that individuals with medical disorder have nonspecific psychological distress, uncomfortable bodily sensations, and treatment side effect.

The odds of participants with common mental disorders to have insomnia were nearly nine times the odds of those without those psychiatric disorders. This finding was supported by different studies done in Nigeria [9], USA [15], UK [17], Sweden [18], and Canada [23]. The elevated risk for psychological distress in insomniac's could be explained by sharing the same risk factors for insomnia and may be because mentally ill individuals have poorer psychosocial functioning and greater deterioration in life.

Excessive noise at bed time was associated with insomnia with the odds of two times and the possible reason for this association may be because noise interrupts normal physiology of the sleep process that was supported by a study done in Portugal [19].

## 5. Conclusion

Insomnia is highly prevalent in Dessie town, Ethiopia, and associated with many factors; older age, female gender, single and widowed by marital status, current substance use (khat, cigarette, and caffeinated beverage use), and current medical-surgical disorders, psychological distress, and excessive noise during the night time. A majority of participants with insomnia disorder expressed a need for treatment, indicating a public health problem.

The generalizability of this study is somewhat limited to the study setting, but as this study is the pioneer one to increase the generalizability of this issues, we recommended researchers to study in other parts of the countries.

## 6. Limitation of the Study

The study does not show temporal relationship between insomnia and blamed factors.

(i) Since the study is a cross-sectional descriptive design, it has a limitation to identify and formulate a causal association, as to how and when the associations are established.

The questionnaire used to assess insomnia and common mental disorder is prone to recall bias.

## 7. Strength of the Study

Data of this study were obtained through home-based face-to-face interviews by qualified and trained psychiatric nurses, reliable measurements, and cautious data handling.

The study identifies useful information that will inform policy makers to design a strategy to reduce the prevalence of insomnia that affects health of the general community at large.

## Figures and Tables

**Table 1:** Distribution of respondents by their sociodemographic characteristics (n=840), Dessie town, North East Ethiopia, 2016.

Variables	(N=840)	%
Age		
18-27	379	45.1
28-37	184	21.9
38-47	98	11.7
48 and above	179	21.3

Sex		
Male	337	40.1
Female	503	59.9

Religion		
Orthodox	469	55.8
Muslim	329	39.2
Others*∗*	42	5

Marital status		
Married	325	38.7
Single (unmarried)	323	38.5
Divorced	157	18.6
widowed	35	4.2

Ethnicity		
Amhara	828	98.6
Others^*∗∗*^	12	1.4

Educational Status		
Not educated	130	15.5
Primary	174	20.7
Secondary	295	35.1
Diploma and above	241	28.7

Occupation		
Employed	115	13.7
Private business	172	20.5
Student	171	20.4
Daily labourer	90	10.7
Housewife and jobless	292	34.8

Income		
<750 ETB	381	45.4
750-1250 ETB	156	18.6
>1250 ETB	303	36.0

N.B: ^*∗∗*^others are Afar, Tigre, and Oromo.

^*∗*^Other religions are protestant and catholic.

**Table 2:** Description of substance use factors among Dessie town adult residents, North East Ethiopia, 2016 (n=840).

Characteristics		N	%
current khat use	Yes	242	28.8
	No	598	71.2

current alcohol use	Yes	317	37.7
	No	523	62.3

current tobacco use	Yes	87	10.4
	No	753	89.6

Caffeinated beverage use	Yes	582	69.3
	No	258	30.7

**Table 3:** Factors associated with insomnia among Dessie town adult residents (bivariate and multivariate analysis) (n=840) North East Ethiopia, 2016.

Characteristics		INSOMNIA		COR (95% CI)	AOR (95%)
		YES	No		
Sex	Male	114	223	1.00	1.00
	Female	246	257	1.87 (1.41, 2.49)	2.74(1.77, 4.24)*∗∗∗*
Age (years)	18-27	144	235	1.00	1.00
	28-37	66	118	0.91 (0.63,1.32)	1.24(0.68, 2.25)
	38-47	48	50	1.57 (1.00,2.45)	3.02 (1.47, 6.20)*∗∗*
	48 and above	102	77	2.16 (1.51, 3.10)	4.67(2.32, 9.40)*∗∗∗*
Marital status	Married	110	215	1.00	1.00
	Single	148	175	1.65 (1.20, 2.27)	2.81(1.59, 4.95)*∗∗∗*
	divorced	79	78	1.98 (1.34, 2.92)	1.03 (0.61, 1.75)
	widowed	23	12	3.75 (1.80, 7.81)	4.20 (1.60, 11.01)*∗∗*
Education	Not educated	68	62	1.00	1.00
	primary	63	111	0.52 (0.33,0.82)	0.69 (0.37, 1.29)
	secondary	115	180	0.58(0.38,0.88)	1.12 (0.59, 2.10)
	Diploma and above	114	127	0.82 (0.53, 1.25)	1.29 (0.63, 2.64)
Occupation	Employed	61	54	1.00	1.00
	Privately owned business	84	88	0.85 (0.53, 1.36)	0.92 (0.47, 1.81)
	Student	61	110	0.49 (0.30, 0.80)	0.51 (0.24, 1.08)
	Daily labourer	19	32	0.78 (0.45, 1.35)	0.89 (0.41, 1.96)
	Housewife and Jobless	112	180	0.55 (0.36, 0.85)	0.54 (0.28, 1.08)
Current Khat use	Yes	120	122	1.47(1.09, 1.98)	1.76(1.19, 2.60)*∗∗*
	No	240	358	1.00	1.00
Current alcohol use	Yes	160	157	1.65(1.24, 2.18)	1.19(0.81,1.74)
	No	200	323	1.00	1.00
Current tobacco use	Yes	60	27	3.36(2.08, 5.41)	**3.13(1.64,5.95)** **∗** **∗**
	No	300	453	1.00	1.00
Caffeinated beverage use	Yes	266	316	1.47 (1.09, 1.99)	**1.67 (1.12, 2.49)** **∗**
	No	94	164	1.00	1.00
Leisure time physical activity	Yes	49	40	1.73 (1.11, 2.70)	1.32 (0.72, 2.40)
	No	311	440	1.00	1.00
Current med/surgical illness	Yes	109	60	3.04 (2.14,4.32)	**2.03(1.18, 3.48)** **∗**
	No	251	420	1.00	1.00
Common mental disorders	Yes	191	52	9.30(6.53,13.26)	**8.92(5.93,13.44)** **∗** **∗** **∗**
	No	169	428	1.00	1.00
Medication use	Yes	140	92	2.68 (1.97,3.66)	1.59 (0.97, 2.61)
	No	220	388	1.00	1.00
Noise	Yes	81	46	2.74 (1.85,4.05)	**2.13 (1.20, 3.78)** **∗**
	No	279	434	1.00	1.00
Light	Yes	73	44	2.52 (1.69,3.77)	1.01 (0.57, 1.81)
	No	287	436	1.00	1.00
Shift work	Yes	79	82	1.37 (0.97, 1.93)	1.21 (0.71, 2.07)
	No	281	398	1.00	1.00
Smart phone use	Yes	142	158	1.33 (0.99, 1.76)	**1**.36 (0.88, 2.09)
	No	218	322	1.00	1.00

1.00 –References, *∗∗* =P value less than 0.01, *∗∗∗* = p-value less than 0.001, and *∗* =P value less than 0.05.

## Data Availability

The data used to support the findings of this study are included within the article.

## Acronyms

AOR: Adjusted odds ratio
AIS: Athens insomnia scale
CI: Confidence interval
DSM-IV-TR: Diagnostic and Statistical Manual of Mental Disorders 4th edition
USA: United States of America
UK: United Kingdom.
